# Diaqua­bis­(hydrogen tartrato)cobalt(II) dihydrate

**DOI:** 10.1107/S1600536811054390

**Published:** 2012-01-07

**Authors:** Chao-Jun Du, Qun-An Zhang, Li-Sheng Wang, Chao-Ling Du

**Affiliations:** a Department of Chemical and Biochemical Engineering, Nanyang Institute of Technology, 473004 Nanyang, Henan, People’s Republic of China; b School of Chemical Engineering and Environment, Beijing Institute of Technology, 100081 Beijing, People’s Republic of China; c College of Science, Nanjing University of Aeronautics and Astronautics, 211100 Nanjing, People’s Republic of China

## Abstract

The title complex, [Co(C_4_H_5_O_6_)_2_(H_2_O)_2_]·2H_2_O, contains a Co^II^ ion, two single deprotonated tartrate anions, two coordinated water mol­ecules and two lattice water mol­ecules. The coordination geometry of the Co^II^ ion is a distorted octa­hedron with two O atoms from two coordinated water mol­ecules occupying *cis* positions in the equatorial plane and four O atoms from two hydrogen tartrate ions occupying the remaining positions. In the crystal, inter­molecular O—H⋯O hydrogen bonds link the mol­ecules into a three-dimensional network.

## Related literature

For general background to chirality, see: Crassous (2009 ▶). For coordination modes of the tartrate anion, see: Al-Dajani *et al.* (2010 ▶); Li *et al.* (2004 ▶). Zhou *et al.* (2006 ▶). For chiral diaqua­bis­(hydrogen tartrato)cobalt(II) dihydrat, see: Yashima *et al.* (2004 ▶).
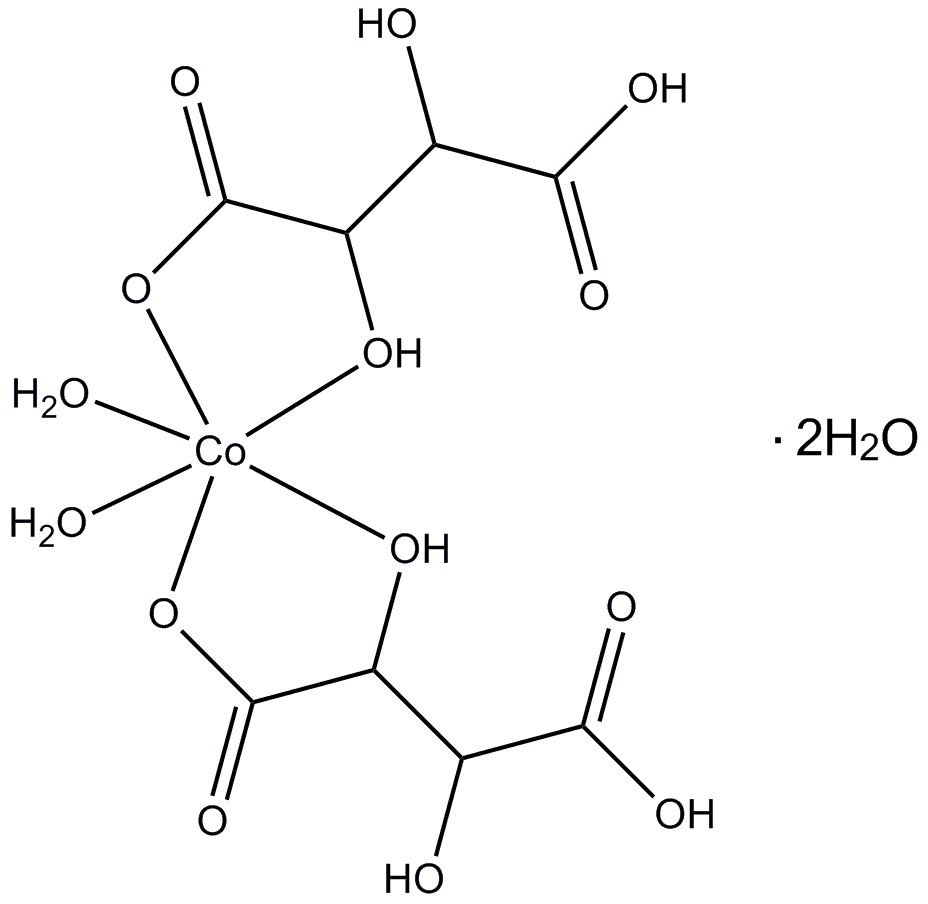


## Experimental

### 

#### Crystal data

[Co(C_4_H_5_O_6_)_2_(H_2_O)_2_]·2H_2_O
*M* *_r_* = 429.15
Orthorhombic, 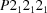

*a* = 7.166 (2) Å
*b* = 7.643 (2) Å
*c* = 27.802 (9) Å
*V* = 1522.7 (8) Å^3^
*Z* = 4
Mo *K*α radiation
μ = 1.22 mm^−1^
*T* = 296 K
0.28 × 0.19 × 0.12 mm

#### Data collection

Bruker APEXII CCD diffractometer
Absorption correction: multi-scan (*SADABS*; Sheldrick, 2008 ▶) *T*_min_ = 0.758, *T*_max_ = 0.864
7644 measured reflections
2705 independent reflections
2465 reflections with *I* > 2σ(*I*)
*R*_int_ = 0.071

#### Refinement

*R*[*F* ^2^ > 2σ(*F* ^2^)] = 0.050
*wR*(*F* ^2^) = 0.116
*S* = 1.03
2705 reflections
227 parameters
H-atom parameters constrained
Δρ_max_ = 0.79 e Å^−3^
Δρ_min_ = −0.53 e Å^−3^
Absolute structure: Flack (1983 ▶), 1303 Friedel pairs
Flack parameter: −0.02 (2)

### 

Data collection: *APEX2* (Bruker, 2008 ▶); cell refinement: *SAINT* (Bruker, 2008 ▶); data reduction: *SAINT*; program(s) used to solve structure: *SHELXS97* (Sheldrick, 2008 ▶); program(s) used to refine structure: *SHELXL97* (Sheldrick, 2008 ▶); molecular graphics: *SHELXTL* (Sheldrick, 2008 ▶); software used to prepare material for publication: *SHELXTL*.

## Supplementary Material

Crystal structure: contains datablock(s) I, global. DOI: 10.1107/S1600536811054390/zj2043sup1.cif

Structure factors: contains datablock(s) I. DOI: 10.1107/S1600536811054390/zj2043Isup2.hkl

Additional supplementary materials:  crystallographic information; 3D view; checkCIF report

## Figures and Tables

**Table 1:** Selected bond lengths (Å)

Co1—O7	2.013 (3)
Co1—O13	2.043 (3)
Co1—O1	2.045 (3)
Co1—O3	2.087 (3)
Co1—O14	2.093 (3)
Co1—O9	2.201 (3)

**Table 2:** Hydrogen-bond geometry (Å, °)

*D*—H⋯*A*	*D*—H	H⋯*A*	*D*⋯*A*	*D*—H⋯*A*
O15—H15*A*⋯O2^i^	0.89	1.96	2.682 (4)	137
O13—H13*A*⋯O11^ii^	0.81	2.20	2.982 (5)	161
O16—H16*B*⋯O8^iii^	0.88	2.34	2.752 (5)	109
O13—H13*B*⋯O15^iv^	0.82	1.88	2.702 (5)	174
O16—H16*A*⋯O5^v^	0.89	1.90	2.757 (5)	161
O11—H11⋯O8^vi^	0.81	1.73	2.542 (4)	171
O6—H6*A*⋯O2^vii^	0.82	2.58	3.269 (5)	143
O6—H6*A*⋯O1^vii^	0.82	1.86	2.648 (4)	160
O14—H14*B*⋯O16^viii^	0.82	1.97	2.796 (5)	174
O14—H14*A*⋯O4^viii^	0.82	2.20	2.934 (4)	149
O3—H3*A*⋯O15^viii^	0.82	1.82	2.629 (4)	166
O15—H15*B*⋯O12	0.89	1.97	2.834 (5)	166
O10—H10⋯O5	0.82	2.13	2.929 (5)	165
O9—H9⋯O16	0.82	1.85	2.631 (4)	160
O4—H4⋯O3	0.82	2.42	2.876 (5)	116
O4—H4⋯O9	0.82	2.39	3.123 (5)	149

Symmetry codes: (i) 
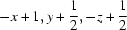
; (ii) 
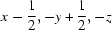
; (iii) 
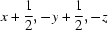
; (iv) 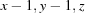; (v) 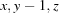; (vi) 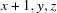; (vii) 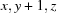; (viii) 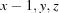.

**Fig. 1.**
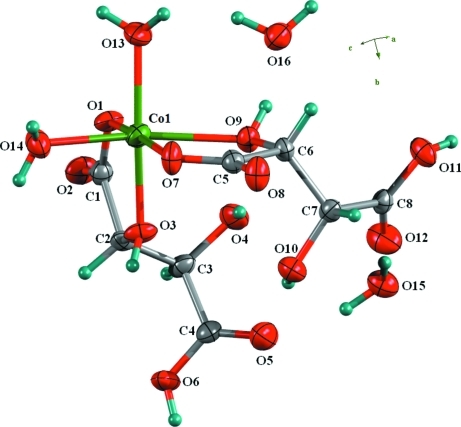
The molecular structure of the title compound, showing 50% probability displacement ellipsoids and the atom-numbering scheme.

**Fig. 2.**
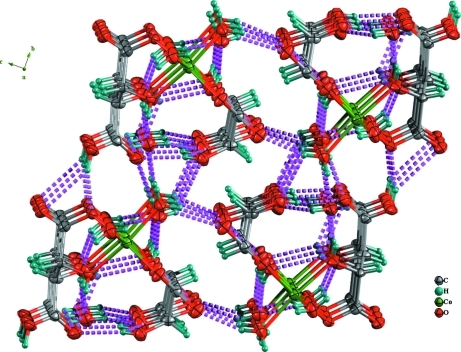
three-dimensional hydrogen-bonded (dashed lines) network of the title compound.

## supplementary crystallographic information

### Comment

Chirality is a signature of life, of biological molecules, and of various inert objects (corkscrew etc). In chemistry, it is expressed not only at the molecular level but also at the supramolecular level and in materials. Chirality in materials involve the dissymmetric arrangement of molecules in a noncovalent assembly (Crassous, 2009). L-Tartaric acid is a simple and cheap chiral ligand source. In the title chiral cobalt(II) complex (scheme 1), the hydroxy and carboxyl group of the tartrate monoanion form a chelate coordination to the Co^II^ atom, this is an unusual coordination mode for the tartrate anion (Al-Dajani *et al.*, 2010); Li *et al.*, 2004; Zhou *et al.*, 2006). Chiral diaquabis(hydrogen tartrato)cobalt(II) dihydrate crystals formed by intermolecular O—H···O hydrogen bonds supramolecular sssembly chiral amplification (Yashima *et al.*, 2004).

The zero-dimensional molecular structure of the title compound is illustrated in Fig. 1. The six-coordinated Co^II^ atom is surrounded by two tartrate monoanions and two water molecules in a distorted octahedral geometry. Two water molecules coordinate to the Co^II^ atom in a *cis* configuration with a normal O13—Co1—O14 bond angle [90.89 (14)]. Two tartrate monoanions chelate to the Co^II^ atom with an unusual coordination mode. the hydroxy O atom and one O atom of the carboxyl group are involved in the chelate bonding but other O atoms are uncoordinated in each ligand. Thus, the carboxyl group binds in a monodentate manner to the Co^II^ atom.

The complex hydrogen-bond network is illustrated in Fig. 2. The hydrogen-bond donors (O3, O4, O6, O9, O10, O11, O13, O14, O15 and O16) from coordinated and uncoordinated hydroxy group, uncoordinated carboxyl group, coordinated and uncoordinated water molecules are connected to neiboring O hydrogen-bond acceptors (Table 2) to form a three dimension infinate network.

### Experimental

L-Tartaric acid (0.04 mol) was dissolved in 50 ml distilled water in a flat bottom flask with magnetic stirrer. Co(CH_3_COO)_2_ (0.02 mol) was added in small portions with continuous stirring for three hours at room temperature. Filtration to obtain clear pink solution after addtion two hours stir. The pink signal crystals suitable for X-ray analysis were obtained within one week by slow evaporation of the filtrate solution. Anal. yield: *ca* 78.6%.

### Refinement

All H atoms were placed in idealized positions (C—H = 0.98 Å, O—H = 0.82 and 0.89 Å), and constrained to ride on the atom to which they are bonded, and were included in the refinement in the riding-model approximation. *U*_iso_(H) values were set equal to 1.2*U*_eq_(parent atom) for methine and 1.5*U*_eq_(parent atom) for all other H atoms.

### Crystal data

**Table:**

[Co(C_4_H_5_O_6_)_2_(H_2_O)_2_]·2H_2_O	*F*(000) = 884
*M**_r_* = 429.15	*D*_x_ = 1.872 Mg m^−^^3^
Orthorhombic, *P*2_1_2_1_2_1_	Mo *K*α radiation, λ = 0.71073 Å
Hall symbol: P 2ac 2ab	Cell parameters from 3026 reflections
*a* = 7.166 (2) Å	θ = 2.8–24.7°
*b* = 7.643 (2) Å	µ = 1.22 mm^−^^1^
*c* = 27.802 (9) Å	*T* = 296 K
*V* = 1522.7 (8) Å^3^	Needle, pink
*Z* = 4	0.28 × 0.19 × 0.12 mm

### Data collection

**Table:**

Bruker APEXII CCD diffractometer	2705 independent reflections
Radiation source: fine-focus sealed tube	2465 reflections with *I* > 2σ(*I*)
graphite	*R*_int_ = 0.071
φ and ω scans	θ_max_ = 25.2°, θ_min_ = 1.5°
Absorption correction: multi-scan (*SADABS*; Sheldrick, 2008)	*h* = −7→8
*T*_min_ = 0.758, *T*_max_ = 0.864	*k* = −9→6
7644 measured reflections	*l* = −27→33

### Refinement

**Table:**

Refinement on *F*^2^	Secondary atom site location: difference Fourier map
Least-squares matrix: full	Hydrogen site location: inferred from neighbouring sites
*R*[*F*^2^ > 2σ(*F*^2^)] = 0.050	H-atom parameters constrained
*wR*(*F*^2^) = 0.116	*w* = 1/[σ^2^(*F*_o_^2^) + (0.0711*P*)^2^ + 0.163*P*] where *P* = (*F*_o_^2^ + 2*F*_c_^2^)/3
*S* = 1.03	(Δ/σ)_max_ < 0.001
2705 reflections	Δρ_max_ = 0.79 e Å^−^^3^
227 parameters	Δρ_min_ = −0.52 e Å^−^^3^
0 restraints	Absolute structure: Flack (1983), 1303 Friedel pairs
Primary atom site location: structure-invariant direct methods	Flack parameter: −0.02 (2)

### Special details

**Table:**

Geometry. All e.s.d.'s (except the e.s.d. in the dihedral angle between two l.s. planes) are estimated using the full covariance matrix. The cell e.s.d.'s are taken into account individually in the estimation of e.s.d.'s in distances, angles and torsion angles; correlations between e.s.d.'s in cell parameters are only used when they are defined by crystal symmetry. An approximate (isotropic) treatment of cell e.s.d.'s is used for estimating e.s.d.'s involving l.s. planes.
Refinement. Refinement of *F*^2^ against ALL reflections. The weighted *R*-factor *wR* and goodness of fit *S* are based on *F*^2^, conventional *R*-factors *R* are based on *F*, with *F* set to zero for negative *F*^2^. The threshold expression of *F*^2^ > σ(*F*^2^) is used only for calculating *R*-factors(gt) *etc*. and is not relevant to the choice of reflections for refinement. *R*-factors based on *F*^2^ are statistically about twice as large as those based on *F*, and *R*- factors based on ALL data will be even larger.

### Fractional atomic coordinates and isotropic or equivalent isotropic displacement parameters (Å^2^)

**Table:**

	*x*	*y*	*z*	*U*_iso_*/*U*_eq_	
C1	0.2015 (6)	0.2982 (6)	0.22522 (15)	0.0254 (10)	
C2	0.1659 (6)	0.4900 (5)	0.21248 (15)	0.0218 (9)	
H2	0.0878	0.5444	0.2373	0.026*	
C3	0.3528 (7)	0.5816 (6)	0.21019 (17)	0.0285 (10)	
H3	0.4092	0.5800	0.2423	0.034*	
C4	0.3299 (6)	0.7714 (6)	0.19428 (15)	0.0271 (9)	
C5	0.0059 (6)	0.4188 (6)	0.03795 (15)	0.0261 (9)	
C6	0.2193 (6)	0.4079 (6)	0.04133 (15)	0.0232 (9)	
H6	0.2664	0.3277	0.0166	0.028*	
C7	0.3000 (6)	0.5901 (6)	0.03307 (16)	0.0253 (9)	
H7	0.2645	0.6281	0.0007	0.030*	
C8	0.5121 (6)	0.5873 (5)	0.03585 (14)	0.0232 (9)	
H3A	0.0017	0.5813	0.1648	0.035*	
H4	0.4078	0.4956	0.1535	0.035*	
H9	0.3470	0.2682	0.0832	0.035*	
H10	0.2862	0.7281	0.0904	0.035*	
H11	0.6988	0.4788	0.0020	0.035*	
H6A	0.2171	0.9701	0.2211	0.035*	
H13A	0.0304	−0.0108	0.0755	0.035*	
H14A	−0.2921	0.3064	0.1762	0.035*	
H15A	0.7561	0.7842	0.1665	0.035*	
H16A	0.4457	0.0121	0.1144	0.035*	
H13B	−0.0403	−0.0611	0.1165	0.035*	
H14B	−0.3154	0.1921	0.1426	0.035*	
H15B	0.7710	0.7047	0.1196	0.035*	
H16B	0.5854	0.0341	0.0756	0.035*	
Co1	0.02672 (7)	0.26348 (7)	0.13168 (2)	0.02417 (18)	
O1	0.1601 (5)	0.1847 (4)	0.19297 (11)	0.0281 (7)	
O2	0.2731 (5)	0.2651 (5)	0.26366 (11)	0.0395 (8)	
O3	0.0751 (4)	0.4992 (4)	0.16748 (12)	0.0301 (7)	
O4	0.4745 (5)	0.4932 (4)	0.17751 (14)	0.0390 (8)	
O5	0.3913 (6)	0.8243 (5)	0.15682 (13)	0.0442 (9)	
O6	0.2315 (5)	0.8642 (4)	0.22412 (12)	0.0401 (9)	
O7	−0.0902 (4)	0.3667 (4)	0.07226 (11)	0.0313 (7)	
O8	−0.0595 (4)	0.4802 (5)	−0.00041 (12)	0.0336 (8)	
O9	0.2674 (4)	0.3434 (4)	0.08767 (11)	0.0271 (7)	
O10	0.2240 (5)	0.7088 (4)	0.06610 (12)	0.0363 (8)	
O11	0.5863 (4)	0.4941 (5)	0.00214 (12)	0.0321 (7)	
O12	0.5946 (5)	0.6683 (5)	0.06641 (14)	0.0446 (9)	
O13	0.0226 (5)	0.0162 (4)	0.10381 (12)	0.0407 (8)	
O14	−0.2372 (4)	0.2264 (5)	0.16226 (11)	0.0369 (8)	
O15	0.8363 (4)	0.7463 (4)	0.14422 (11)	0.0352 (7)	
O16	0.5099 (5)	0.0867 (4)	0.09600 (12)	0.0349 (7)	

### Atomic displacement parameters (Å^2^)

**Table:**

	*U*^11^	*U*^22^	*U*^33^	*U*^12^	*U*^13^	*U*^23^
C1	0.024 (2)	0.026 (2)	0.026 (2)	0.0020 (17)	0.0005 (17)	0.0039 (18)
C2	0.026 (2)	0.020 (2)	0.020 (2)	0.0041 (19)	0.0001 (17)	0.0012 (16)
C3	0.029 (2)	0.021 (2)	0.036 (3)	0.001 (2)	−0.004 (2)	0.0036 (19)
C4	0.028 (2)	0.022 (2)	0.032 (2)	−0.006 (2)	−0.0024 (18)	−0.0023 (19)
C5	0.024 (2)	0.027 (2)	0.027 (2)	0.000 (2)	0.0022 (18)	−0.0007 (17)
C6	0.019 (2)	0.027 (2)	0.024 (2)	0.0020 (18)	0.0022 (17)	0.0001 (18)
C7	0.021 (2)	0.025 (2)	0.030 (2)	0.0005 (19)	−0.0007 (18)	0.0039 (18)
C8	0.022 (2)	0.024 (2)	0.024 (2)	0.0007 (19)	−0.0002 (18)	0.0036 (16)
Co1	0.0261 (3)	0.0232 (3)	0.0232 (3)	−0.0017 (2)	−0.0017 (2)	0.0011 (2)
O1	0.0384 (17)	0.0187 (15)	0.0273 (16)	−0.0002 (14)	−0.0046 (14)	0.0008 (12)
O2	0.0569 (19)	0.0334 (18)	0.0281 (17)	0.0037 (19)	−0.0089 (15)	0.0074 (15)
O3	0.0357 (17)	0.0195 (14)	0.0351 (18)	0.0060 (14)	−0.0136 (14)	0.0015 (13)
O4	0.0287 (16)	0.0321 (16)	0.056 (2)	0.0060 (17)	0.0059 (16)	−0.0032 (15)
O5	0.061 (2)	0.0341 (18)	0.037 (2)	0.0042 (18)	0.0137 (18)	0.0076 (15)
O6	0.060 (2)	0.0207 (16)	0.039 (2)	0.0093 (17)	0.0176 (18)	0.0007 (14)
O7	0.0240 (15)	0.046 (2)	0.0241 (17)	−0.0022 (15)	0.0036 (13)	0.0092 (14)
O8	0.0224 (16)	0.050 (2)	0.0282 (17)	−0.0008 (16)	−0.0040 (13)	0.0106 (15)
O9	0.0245 (15)	0.0301 (16)	0.0267 (16)	0.0037 (14)	0.0003 (13)	0.0065 (12)
O10	0.0374 (17)	0.0328 (19)	0.0388 (19)	0.0030 (15)	0.0070 (15)	−0.0054 (15)
O11	0.0205 (15)	0.0425 (19)	0.0334 (18)	0.0038 (15)	−0.0002 (13)	−0.0079 (15)
O12	0.0339 (18)	0.050 (2)	0.050 (2)	−0.0003 (18)	−0.0088 (17)	−0.0170 (18)
O13	0.059 (2)	0.0296 (16)	0.0334 (18)	−0.0025 (19)	0.0121 (18)	−0.0087 (13)
O14	0.0297 (15)	0.0430 (19)	0.0380 (17)	−0.0017 (17)	0.0082 (13)	−0.0089 (16)
O15	0.0335 (14)	0.0378 (18)	0.0343 (17)	0.0057 (18)	−0.0044 (13)	−0.0027 (15)
O16	0.0340 (17)	0.0323 (16)	0.0383 (18)	−0.0030 (16)	0.0010 (15)	0.0030 (13)

### Geometric parameters (Å, °)

**Table:**

C1—O2	1.212 (5)	C8—O11	1.292 (5)
C1—O1	1.282 (5)	Co1—O7	2.013 (3)
C1—C2	1.530 (6)	Co1—O13	2.043 (3)
C2—O3	1.412 (5)	Co1—O1	2.045 (3)
C2—C3	1.513 (6)	Co1—O3	2.087 (3)
C2—H2	0.9800	Co1—O14	2.093 (3)
C3—O4	1.429 (6)	Co1—O9	2.201 (3)
C3—C4	1.525 (6)	O3—H3A	0.8221
C3—H3	0.9800	O4—H4	0.8224
C4—O5	1.201 (5)	O6—H6A	0.8200
C4—O6	1.300 (5)	O9—H9	0.8195
C5—O7	1.242 (5)	O10—H10	0.8215
C5—O8	1.256 (5)	O11—H11	0.8148
C5—C6	1.534 (6)	O13—H13A	0.8150
C6—O9	1.422 (5)	O13—H13B	0.8225
C6—C7	1.526 (6)	O14—H14A	0.8240
C6—H6	0.9800	O14—H14B	0.8243
C7—O10	1.401 (5)	O15—H15A	0.8923
C7—C8	1.522 (6)	O15—H15B	0.8877
C7—H7	0.9800	O16—H16A	0.8926
C8—O12	1.207 (5)	O16—H16B	0.8809
			
O2—C1—O1	125.0 (4)	O7—Co1—O13	92.59 (14)
O2—C1—C2	118.4 (4)	O7—Co1—O1	173.65 (13)
O1—C1—C2	116.6 (4)	O13—Co1—O1	92.89 (13)
O3—C2—C3	110.4 (3)	O7—Co1—O3	97.03 (13)
O3—C2—C1	109.3 (3)	O13—Co1—O3	169.06 (14)
C3—C2—C1	107.8 (3)	O1—Co1—O3	77.23 (12)
O3—C2—H2	109.8	O7—Co1—O14	90.58 (13)
C3—C2—H2	109.8	O13—Co1—O14	90.89 (14)
C1—C2—H2	109.8	O1—Co1—O14	92.53 (13)
O4—C3—C2	110.4 (4)	O3—Co1—O14	94.21 (13)
O4—C3—C4	109.3 (4)	O7—Co1—O9	76.20 (12)
C2—C3—C4	110.9 (4)	O13—Co1—O9	93.28 (13)
O4—C3—H3	108.7	O1—Co1—O9	100.29 (12)
C2—C3—H3	108.7	O3—Co1—O9	84.00 (13)
C4—C3—H3	108.7	O14—Co1—O9	166.29 (12)
O5—C4—O6	124.7 (4)	C1—O1—Co1	119.5 (3)
O5—C4—C3	122.2 (4)	C2—O3—Co1	117.1 (2)
O6—C4—C3	113.1 (4)	C2—O3—H3A	114.3
O7—C5—O8	124.4 (4)	Co1—O3—H3A	120.7
O7—C5—C6	119.2 (4)	C3—O4—H4	98.7
O8—C5—C6	116.4 (4)	C4—O6—H6A	122.9
O9—C6—C7	111.1 (3)	C5—O7—Co1	121.7 (3)
O9—C6—C5	108.4 (3)	C6—O9—Co1	114.2 (2)
C7—C6—C5	108.6 (4)	C6—O9—H9	106.0
O9—C6—H6	109.5	Co1—O9—H9	115.8
C7—C6—H6	109.5	C7—O10—H10	116.3
C5—C6—H6	109.5	C8—O11—H11	119.3
O10—C7—C8	111.4 (4)	Co1—O13—H13A	126.8
O10—C7—C6	110.2 (3)	Co1—O13—H13B	120.7
C8—C7—C6	111.0 (4)	H13A—O13—H13B	105.6
O10—C7—H7	108.1	Co1—O14—H14A	121.4
C8—C7—H7	108.1	Co1—O14—H14B	112.8
C6—C7—H7	108.1	H14A—O14—H14B	102.9
O12—C8—O11	126.3 (4)	H15A—O15—H15B	108.1
O12—C8—C7	121.2 (4)	H16A—O16—H16B	113.2
O11—C8—C7	112.5 (4)		
			
O2—C1—C2—O3	−177.5 (4)	C2—C1—O1—Co1	−6.6 (5)
O1—C1—C2—O3	5.5 (5)	O13—Co1—O1—C1	179.3 (3)
O2—C1—C2—C3	62.5 (5)	O3—Co1—O1—C1	4.1 (3)
O1—C1—C2—C3	−114.4 (4)	O14—Co1—O1—C1	−89.7 (3)
O3—C2—C3—O4	−64.4 (4)	O9—Co1—O1—C1	85.4 (3)
C1—C2—C3—O4	54.8 (5)	C3—C2—O3—Co1	116.2 (3)
O3—C2—C3—C4	56.9 (5)	C1—C2—O3—Co1	−2.2 (4)
C1—C2—C3—C4	176.2 (4)	O7—Co1—O3—C2	−177.8 (3)
O4—C3—C4—O5	7.5 (6)	O13—Co1—O3—C2	−26.5 (9)
C2—C3—C4—O5	−114.4 (5)	O1—Co1—O3—C2	−0.6 (3)
O4—C3—C4—O6	−175.4 (4)	O14—Co1—O3—C2	91.0 (3)
C2—C3—C4—O6	62.7 (5)	O9—Co1—O3—C2	−102.6 (3)
O7—C5—C6—O9	−3.1 (6)	O8—C5—O7—Co1	178.2 (3)
O8—C5—C6—O9	177.3 (4)	C6—C5—O7—Co1	−1.3 (6)
O7—C5—C6—C7	−124.0 (4)	O13—Co1—O7—C5	−89.3 (4)
O8—C5—C6—C7	56.4 (5)	O3—Co1—O7—C5	85.4 (4)
O9—C6—C7—O10	−62.8 (4)	O14—Co1—O7—C5	179.8 (3)
C5—C6—C7—O10	56.4 (4)	O9—Co1—O7—C5	3.4 (3)
O9—C6—C7—C8	61.0 (4)	C7—C6—O9—Co1	124.9 (3)
C5—C6—C7—C8	−179.8 (3)	C5—C6—O9—Co1	5.6 (4)
O10—C7—C8—O12	6.1 (6)	O7—Co1—O9—C6	−5.0 (3)
C6—C7—C8—O12	−117.0 (5)	O13—Co1—O9—C6	86.9 (3)
O10—C7—C8—O11	−172.6 (3)	O1—Co1—O9—C6	−179.6 (3)
C6—C7—C8—O11	64.2 (5)	O3—Co1—O9—C6	−103.8 (3)
O2—C1—O1—Co1	176.7 (3)	O14—Co1—O9—C6	−20.7 (7)

### Hydrogen-bond geometry (Å, °)

**Table:**

*D*—H···*A*	*D*—H	H···*A*	*D*···*A*	*D*—H···*A*
O15—H15A···O2^i^	0.89	1.96	2.682 (4)	137
O13—H13A···O11^ii^	0.81	2.20	2.982 (5)	161
O16—H16B···O8^iii^	0.88	2.34	2.752 (5)	109
O13—H13B···O15^iv^	0.82	1.88	2.702 (5)	174
O16—H16A···O5^v^	0.89	1.90	2.757 (5)	161
O11—H11···O8^vi^	0.81	1.73	2.542 (4)	171
O6—H6A···O2^vii^	0.82	2.58	3.269 (5)	143
O6—H6A···O1^vii^	0.82	1.86	2.648 (4)	160
O14—H14B···O16^viii^	0.82	1.97	2.796 (5)	174
O14—H14A···O4^viii^	0.82	2.20	2.934 (4)	149
O3—H3A···O15^viii^	0.82	1.82	2.629 (4)	166
O15—H15B···O12	0.89	1.97	2.834 (5)	166
O10—H10···O5	0.82	2.13	2.929 (5)	165
O9—H9···O16	0.82	1.85	2.631 (4)	160
O4—H4···O3	0.82	2.42	2.876 (5)	116
O4—H4···O9	0.82	2.39	3.123 (5)	149

Symmetry codes: (i) −*x*+1, *y*+1/2, −*z*+1/2; (ii) *x*−1/2, −*y*+1/2, −*z*; (iii) *x*+1/2, −*y*+1/2, −*z*; (iv) *x*−1, *y*−1, *z*; (v) *x*, *y*−1, *z*; (vi) *x*+1, *y*, *z*; (vii) *x*, *y*+1, *z*; (viii) *x*−1, *y*, *z*.
